# Study of Double-Side Ultrasonic Embossing for Fabrication of Microstructures on Thermoplastic Polymer Substrates

**DOI:** 10.1371/journal.pone.0061647

**Published:** 2013-04-22

**Authors:** Yi Luo, Xu Yan, Na Qi, Xiaodong Wang, Liangjiang Wang

**Affiliations:** Key Laboratory for Micro/Nano Technology and System of Liaoning Province, Dalian University of Technology, Dalian, China; University of California, Berkeley, United States of America

## Abstract

Double-side replication of polymer substrates is beneficial to the design and the fabrication of 3-demensional devices. The ultrasonic embossing method is a promising, high efficiency and low cost replication method for thermoplastic substrates. It is convenient to apply silicon molds in ultrasonic embossing, because microstructures can be easily fabricated on silicon wafers with etching techniques. To reduce the risk of damaging to silicon molds and to improve the replication uniformity on both sides of the polymer substrates, thermal assisted ultrasonic embossing method was proposed and tested. The processing parameters for the replication of polymethyl methacrylate (PMMA), including ultrasonic amplitude, ultrasonic force, ultrasonic time, and thermal assisted temperature were studied using orthogonal array experiments. The influences of the substrate thickness, pattern style and density were also investigated. The experiment results show that the principal parameters for the upper and lower surface replication are ultrasonic amplitude and thermal assisted temperature, respectively. As to the replication uniformity on both sides, the ultrasonic force has the maximal influence. Using the optimized parameters, the replication rate reached 97.5% on both sides of the PMMA substrate, and the cycle time was less than 50 s.

## Introduction

Thermoplastic polymer materials play an important role in Micro-electro-mechanical system (MEMS) devices and have been investigated over the past decade. They are widely used in the fields of micro-optics [1], lighting [2], sensors [3], micro total analysis systems(µTAS) [4] and artificial organs [5], etc. Thermoplastic polymer micro devices were successfully developed using materials such as poly(methyl methacrylate)(PMMA) [6], polycarbonate(PC) [7], and cyclo olefin copolymer(COP) [8]. The fabrication methods mainly include injection molding, hot embossing and thermoforming [9].

Compared with other replication methods, injection molding has the shortest cycle time. For fabrication of microstructures, the cycle time is about 1 min, which meets the mass production requirements. But the molten polymer needs to flow through a long and narrow cavity before filling into the microstructures and thus it is difficult to fabricate fine microstructures.

Hot embossing is a straightforward replication method for microstructures. Before embossing, the polymer substrate must be heated above the glass transition temperature (*Tg*), the substrates are unnecessarily softened entirely and the heating process is time-consuming. Usually more than 10 min is needed. To reduce the cycle time, the hot roller embossing [10] was proposed. This process is achieved by transferring the micro patterning to a thin foil from a cylindrical roller mold. However, this method usually needs higher temperature and embossing force, so the mold fabrication is a challenge issue. Meanwhile this method is not suitable for polymer sheet with the thickness over 1 mm.

T. Senn reported, by using HEX03 hot embossing machine, three dimensional structures were hot embossed or thermoformed in polymer foils [9]. Still, this approach is suitable for thin polymers, and the best replication result has been achieved with the thickness of 70 µm.

Ultrasonic vibration has been widely used in thermoplastic welding for decades. In recent years, using ultrasonic vibration to replicate microstructures and to package micro devices were reported. The cycle time is typically less than 1 min.

Ultrasonic vibration has been used in hot embossing as an auxiliary heating source. H. Mekaru et al. reported that using an electroformed Ni mold, micro patterns with different sizes form 100 µm^2^ to 1.2 mm^2^ were replicated on a polycarbonate (PC) sheet. In the ultrasonic embossing process, the PC sheet was heated to a temperature above its *Tg* and the ultrasonic vibration was imposed with the maximum amplitude of 1.8 µm [11].

Ultrasonic embossing process has been further developed to use ultrasonic vibration directly to heat the mold and polymer. S.J. Liu et al. reported using ultrasonic embossing to fabricate double-side surface-relief plastic diffusers [12]. The top mold was directly machined on the vibration horn while the bottom molds were made onto two mild steel sheets. H.W. Yu et al. reported using two types of Ni micromolds to replicate microstructures on PMMA sheet, and the molding time can be reduced to 2–2.5 s [13]. K. Burlage reported using ultrasonic vibration to replicate microstructures on polymer films on single-side or double-side [14]. In single-side replication, the metal mold was used, and in double-side replication, the horn was also structured to make the upper surface of the polymer film patterned. Y.S. Seo et al. reported using a horn with microstructure to replicate different sizes of micro patterns [15].

All these studies show that ultrasonic embossing is an efficient method to fabricate microstructures on polymer substrate. In all these reports, metal molds or patterned horns were used in ultrasonic embossing. Electroplating a metal mold is a time consuming process to reach enough thickness of micro mold and obtain enough strength for embossing. Meanwhile, using precision numerical machine tools to fabricate the micro mold has the limitations on size and quality of microstructures. As another alternative, silicon mold is a good choice. The fabrication process on silicon substrates is widely used in MEMS, either dry etching or wet etching is low cost. However, using silicon mold in embossing introduces a challenge due to its fragile and easily to crack under the ultrasonic vibration.

This paper focused on developing an efficient process to fabricate double-side microstructures on thermoplastic sheet. With the development of large scale integrated micro devices, such as microfluidics, micro-optics, and fabrication of microstructure on double-side of the polymer sheets can benefit the design and the fabrication. Double-side ultrasonic embossing has more fabrication problems than the single-surface embossing process.

The first one is the tradeoff between energy consumption and ultrasonic amplitude. For double-side embossing, the vibration energy consumption is larger than that of the single-surface embossing and thus larger ultrasonic amplitude is needed. However, larger amplitude always means it is difficult to control during the process of embossing. Because the heat generates rapidly under large ultrasonic amplitude, the operation window will be extremely narrow. Moreover, large amplitude also adds the risk of mold crack. The second one is the uniformity of the replication on both surfaces. The ideal double-side replication has almost the same structure fidelity on both sides of the polymer sheet. However, according to the ultrasonic transmitting routine, the upper surface softened first and the softened polymer absorbs more ultrasonic energy, therefore the lower surface received less ultrasonic energy. As a result, using ultrasonic vibration to emboss directly will result in the unequivalence of energy on the two surfaces, thus the replication on upper and lower surface will be different.

In this paper, thermal assisted double-side ultrasonic embossing method is proposed. The polymer substrate is preheated to a temperature of 40–60°C that is below its *Tg*, then applied ultrasonic vibration to form the microstructures. Unlike the ultrasonic vibration as an auxiliary heating source [11], the preheated temperature is far below the *Tg*, thus the ultrasonic vibration induced heat is the main source of the heat in embossing. Meanwhile, the heating process of the polymer can be shortened, too. Only the two surfaces, not the whole bulk of the polymer substrate need preheating. The temperature gap between the initial polymer temperature and the forming temperature of the polymer is reduced to 40–60°C, thus a small amplitude can be used in embossing, which is reduce the risk of the damaging the mold. Furthermore, the temperature on the lower surface is higher than that of the upper surface, which makes it quicker to accumulate enough heat and benefits the replication equivalency on the two surfaces.

A commercialized ultrasonic welding machine was used in experiments in this study, the silicon molds were fabricated using wet etching. The effect of ultrasonic parameters, thermal assisted temperature, pattern style and pattern density on embossing performance were investigated.

## Experiments

### Materials

PMMA substrate (Optix Acrylic Sheet, Asahi Kasei Corporation, Shanghai, China) with thickness of 1 and 2 mm were used in the experiments. The *Tg* of the PMMA was 105°C, and the dimension of the substrates was 32×32 mm.

Silicon molds for double-side ultrasonic embossing were made of n-type silicon (100) wafers (Tianjin Institute for Semiconductor Technology) and using anisotropic wet etching. KOH : IPA : H_2_O = 40 g : 30 ml : 100 ml was used as an etchant at 73°C. The etching time was 20 min, and the depth of the microstructures was 21.3 µm.

### Design of the mold patterns

**Figure 1:**
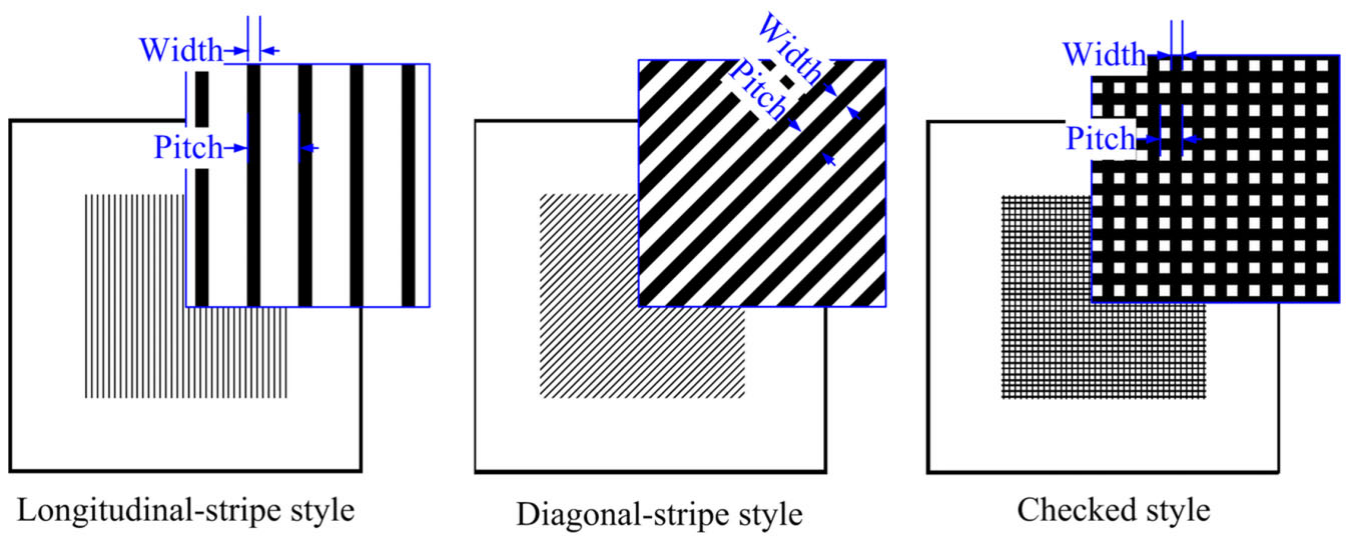
Three patterns on the photo mask for fabricate silicon mold.

There were three types of micro patterns designed on different molds, as shown in Fig. 1. The first one was longitudinal-stripe style, the second one was diagonal-stripe style, and the third one was checked style. The detailed dimensions of different micro patterns are listed in Table 1.

**Table 1:** The dimensions of the micro patterns for different molds.

Item	Longitudinal-stripe style		Diagonal-strip style		Checked style	
Mold number	No. 1	No. 2	No. 3	No. 4	No. 5	No. 6
Pitch (µm)	140	280	560	140	560	140
Width (µm)	70	70	70	70	70	70

### Thermal assisted ultrasonic embossing system setup

The essential components for the thermal assisted ultrasonic embossing facility are ultrasonic generation machine, fixture and hot plate. A 1500 W ultrasonic welding machine (2000×f, Branson Ultrasonics Co.) was used for all the experiments as the ultrasonic generation machine. The output frequency was 30 kHz, and the vibration amplitude was range from 6–60 µm with an increment step of 0.6 µm. The maximum force applied in the ultrasonic and holding stage was both 680 N, and the longest ultrasonic time was 30 s.

**Figure 2:**
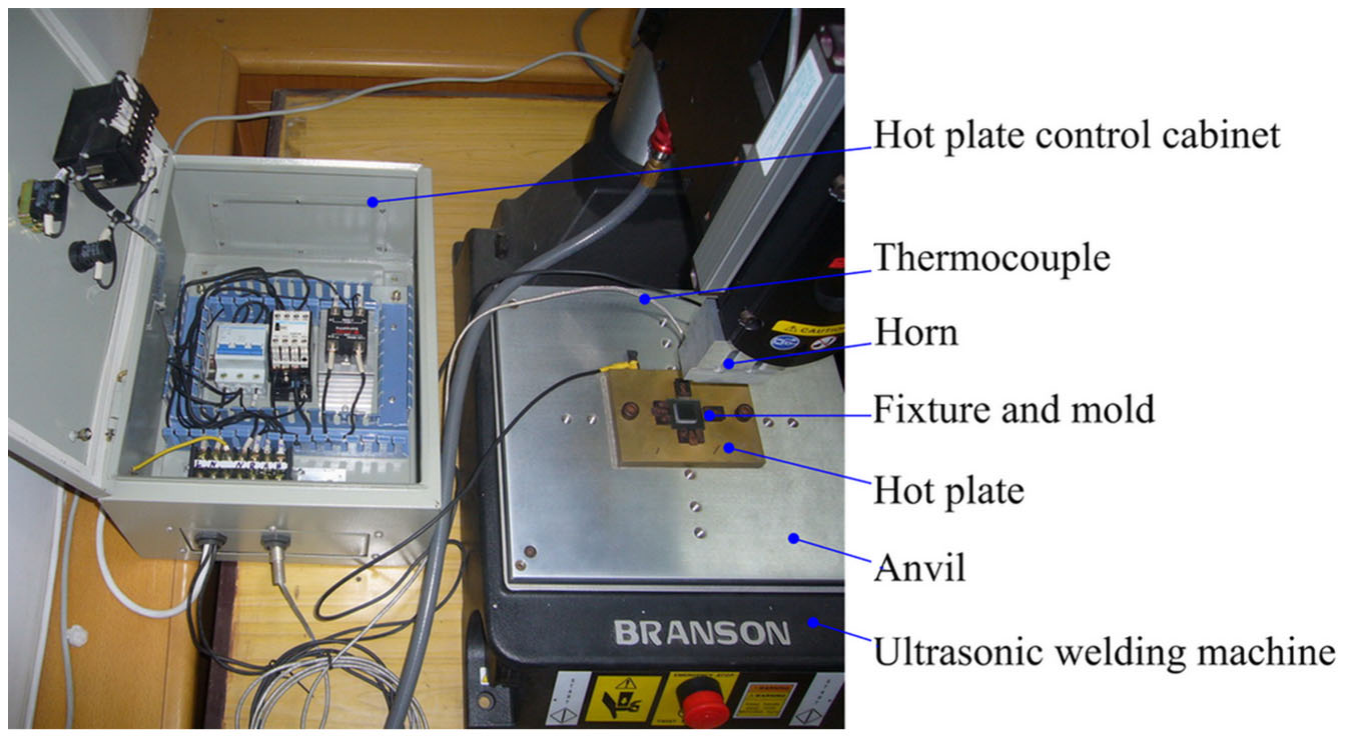
Thermal assisted ultrasonic embossing system.

A steel fixture was customized to suit the dimension of the polymer substrates. A hot plate was mounted on the anvil of the ultrasonic welding machine. It is comprised of a copper hot plate with the heating power of 300 W, a temperature sensor and a temperature control cabinet. The temperature can rise from room temperature to 250°C with the control accuracy of ±1°C. Figure 2 shows the facility of the thermal assisted ultrasonic embossing system.

### Ultrasonic embossing process

**Figure 3:**
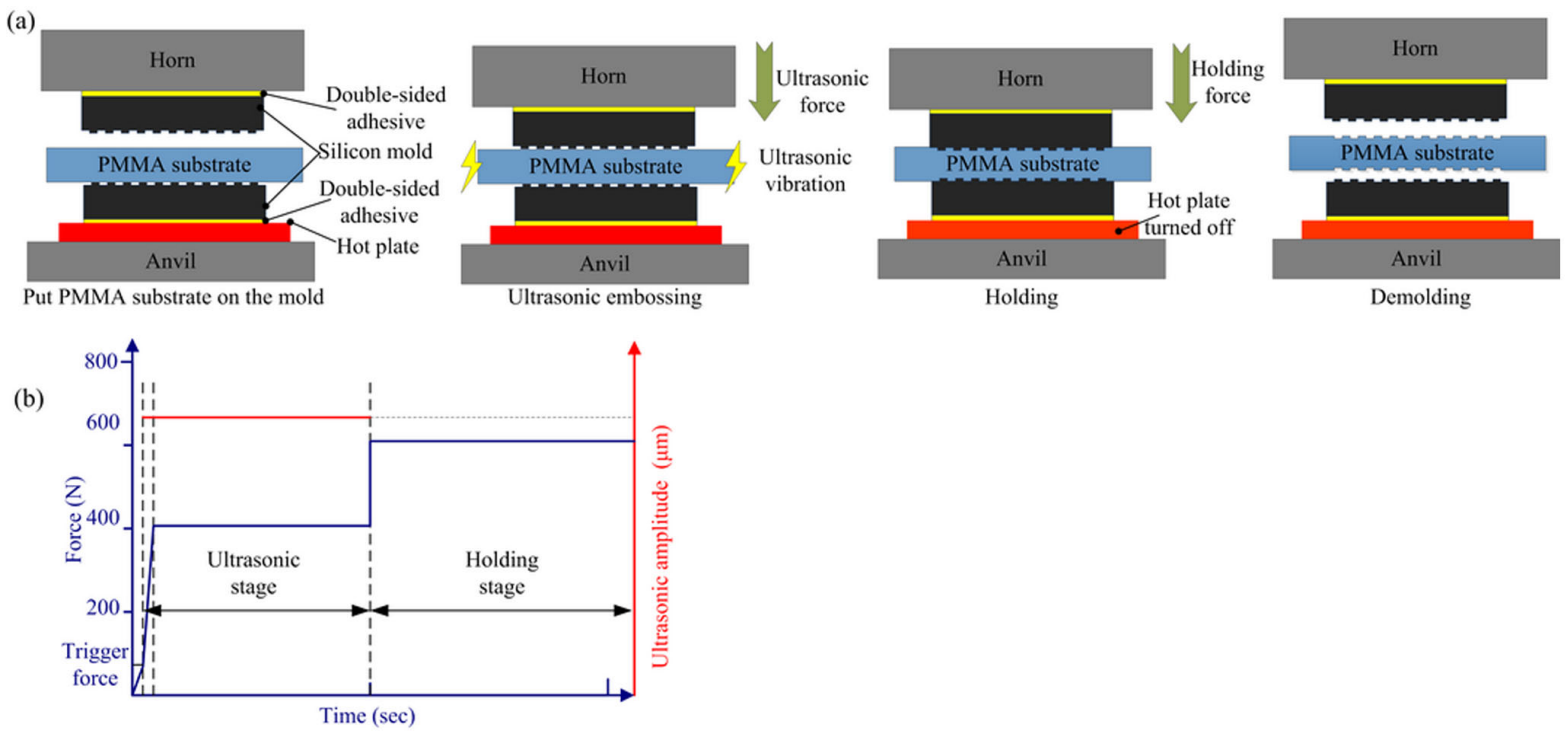
Thermal assisted ultrasonic embossing (a) schematic process; (b) the force and amplitude in an ultrasonic embossing process.

Figure 3 demonstrates the process of the thermal assisted ultrasonic embossing. Figure 3(a) is the assembly for thermal assisted double-side ultrasonic embossing, and Fig. 3(b) outlines the parameter curves of thermal assisted ultrasonic embossing.

The parameters pre-set in the ultrasonic welding machine are trigger force, ultrasonic force, ultrasonic amplitude, ultrasonic time, holding force, and holding time. The silicon molds were placed on the horn and the hot plate, respectively. Silicon molds tend to crack under the ultrasonic vibration for its fragility. The silicon wafer with thickness less than 1 mm cannot be utilized, because the mold life is usually less than 5 times. To avoid damaging the silicon molds, the silicon wafer with thickness of 2 mm was selected in this study. The silicon molds were mounted on the horn and the hot plate by thermal conducted double-sided adhesives (Bond-Ply 100, Bergquist) to reduce the cross slip under the ultrasonic vibration. The arrangement of the silicon molds on the horn and the hot plate was shown in Fig. 3(a). With these improvements, the silicon mold can be used in the ultrasonic embossing without damage.

Firstly, the parameters of ultrasonic welding machine and thermal assisted temperature were set; then the PMMA substrate, which was already preheated on the hot plate, was put between the silicon molds. When the horn went down, the force sensor integrated in the ultrasonic welding machine detecting the force in real time, and when it reached the trigger value, the ultrasonic vibration with the pre-set amplitude was turned on, and the force continued to increase until it reached the value of the ultrasonic force, usually in less than 0.1 s. The polymer at the interface of PMMA - mold was heated by the ultrasonic energy and softened. When the time reached the pre-set ultrasonic time, the vibration turned off and the force changed into holding force. The softened polymer at the interface cooled down under the holding force which helped further filling of the microstructures on the mold. Then the horn lifted, and the embossing process finished.

In order to research the influence of the ultrasonic parameters and thermal assisted temperature to replication results, a L^16^(4^4^) orthogonal arrays with sixteen rows was designed. Ultrasonic amplitude, ultrasonic time and ultrasonic force and thermal assisted temperature were selected as the controllable parameters. The depth of the microstructures on both sides of the PMMA substrates was measured by the surface profiler (ET100, Kosaka). The experimental results are shown in Table 2. In all the experiments, holding force and the holding time were set as 600 N and 25 s, respectively. The holding time was chosen by the temperature testing experiments detailed described in section 2.5. The highest replication rate in experiment No. 14 reached 97.5% on both sides of the PMMA substrate.

**Table 2:** Results of the orthogonal experiments.

No.	Thermal assisted temperature (°C)	Ultrasonic amplitude (µm)	Ultrasonic time (s)	Ultrasonic force (N)	Depth of the micro structures on PMMA substrate (µm)	
					Upper surface	Lower surface
1	50	9.0	19	300	0.18	1.95
2	50	7.8	22	400	0.12	0.28
3	50	9.6	16	350	0.49	6.57
4	50	8.4	25	250	1.75	2.35
5	55	9.0	25	350	20.70	20.75
6	55	7.8	16	250	0.22	0.94
7	55	9.6	22	300	10.52	20.71
8	55	8.4	19	400	0.27	2.38
9	60	7.8	25	300	0.42	6.82
10	60	9.0	16	400	0.57	11.84
11	60	8.4	22	350	2.53	10.05
12	60	9.6	19	250	20.72	20.67
13	65	7.8	19	350	0.56	14.83
14	65	9.0	22	250	20.78	20.77
15	65	8.4	16	300	1.83	14.52
16	65	9.6	25	400	20.72	20.65

### Temperature testing experiments

**Figure 4:**
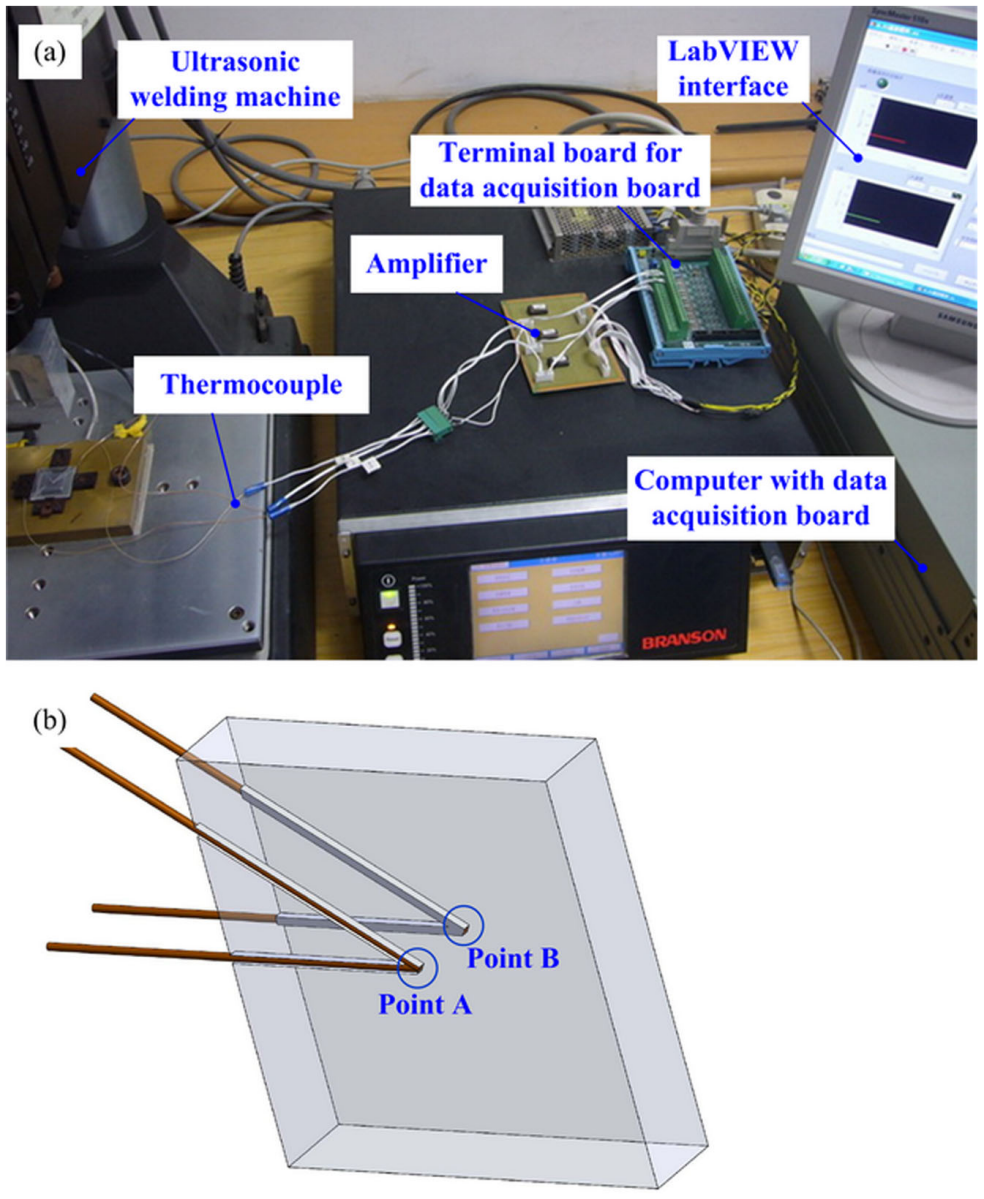
The temperature testing system (a) schematic of the system; (b) position of the thermocouples.

The temperature testing system consists of rapid response thermocouples (Chal-0005, Omega Company, USA), data amplifier (AD524, Analog Devices Inc., USA), multi-channel data acquisition board (NI DAQPad-6015, National Instruments, USA) and industrial computer, as shown in Fig. 4(a). The thermocouples were placed at the center of the upper surface and the lower surface of the PMMA substrate respectively, named as Point A and B, as shown in Fig. 4(b). The thermocouples were buried by three steps. Firstly, use knife to crave out grooves on the PMMA substrate until the depth of the groove was almost the same as the diameter of the thermocouples. Secondly, put the thermocouples into grooves; be sure that the thermocouples were below the surface of the PMMA substrate. Thirdly, connect the thermocouple to a DC power supply (Matrix MPS-3003L-3, Matrix Technology Inc. China) and under the condition of 1.0 A for 2 s. The thermocouple was heated under the current and the heat melted the adjacent PMMA. Therefore, the thermocouple was fixed when the polymer was cooled down.

The parameters were selected as: thermal assisted temperature 65°C, ultrasonic time 22 s, ultrasonic amplitude 9.0 µm, ultrasonic force 250 N, which was the No. 14 experiment in Table 2. The replication experiment was successful as the replication rate reached 97.5% on both sides, which implied that the amount of softened polymer generated by the ultrasonic vibration was large enough to form the microstructures and to choose the holding time based on this situation. The holding force was selected as 600 N, because a large holding force can benefit the forming of the microstructure. Though the maximum holding force can be set is 680 N for the machine, the stability of the output force decreased when the force was approaching it. Therefore, 600 N were selected as the holding force in this study.

**Figure 5:**
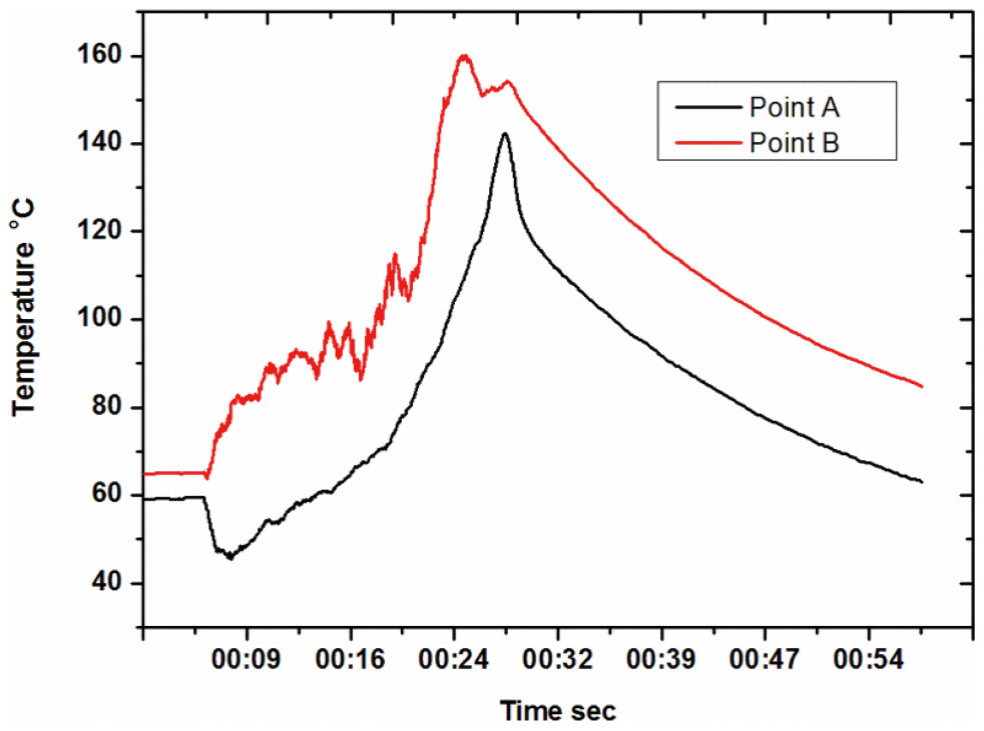
The temperature curve on both sides of the PMMA substrate in embossing.

The temperature curve at the Point A and B is shown in Fig. 5. The temperature at Point B was higher. However, Point A had a larger temperature decreasing speed for the horn, and the upper silicon mold was not preheated, which could transfer the heat more quickly. At the lower surface of PMMA substrate, the heat capacity of the hot plate was large, which resulted in a slow temperature decreasing speed at Point B. Commonly, the PMMA is hard enough and cannot be shaping at 90°C under the holding force of 600 N. The timefrom the ultrasonic vibration stop point to 90°C was 24 s, as shown in Fig. 5. Thus the holding time was chosen as 25 s in orthogonal array experiments.

## Results and Discussions

### The characteristic of the temperature curve for embossing

In Fig. 5, the ultrasonic vibration started at the 6^th^ second and stopped at the 28^th^ second. At the beginning, the temperature at the upper surface had a quick drop then followed by a rise. When the cold upper mold contacted the PMMA substrate, the heat generation rate cannot keep up with the heat dissipation speed, thus the temperature dropped about 15°C from 6^th^ to 8^th^ second. Then the temperature began to rise once the heat generation rate larger than the dissipation speed.

There are two methods to bury the thermocouples, the first one is with the same depth below the surfaces of PMMA substrate, and the second is to make the thermocouples on the lower surface buried a little deeper. We chose the first method, because it can record the real temperature during most of the embossing process. When the polymer on the lower surface reaches 158°C at the 25^th^ second, as show in Fig. 5, the thermocouple contacted the silicon mold and recorded the temperature a little lower than the actual value. In the second method, the temperature from 6^th^ to 25^th^ second would have deviation to the actual one.

### Effects of the embossing parameters

**Figure 6:**
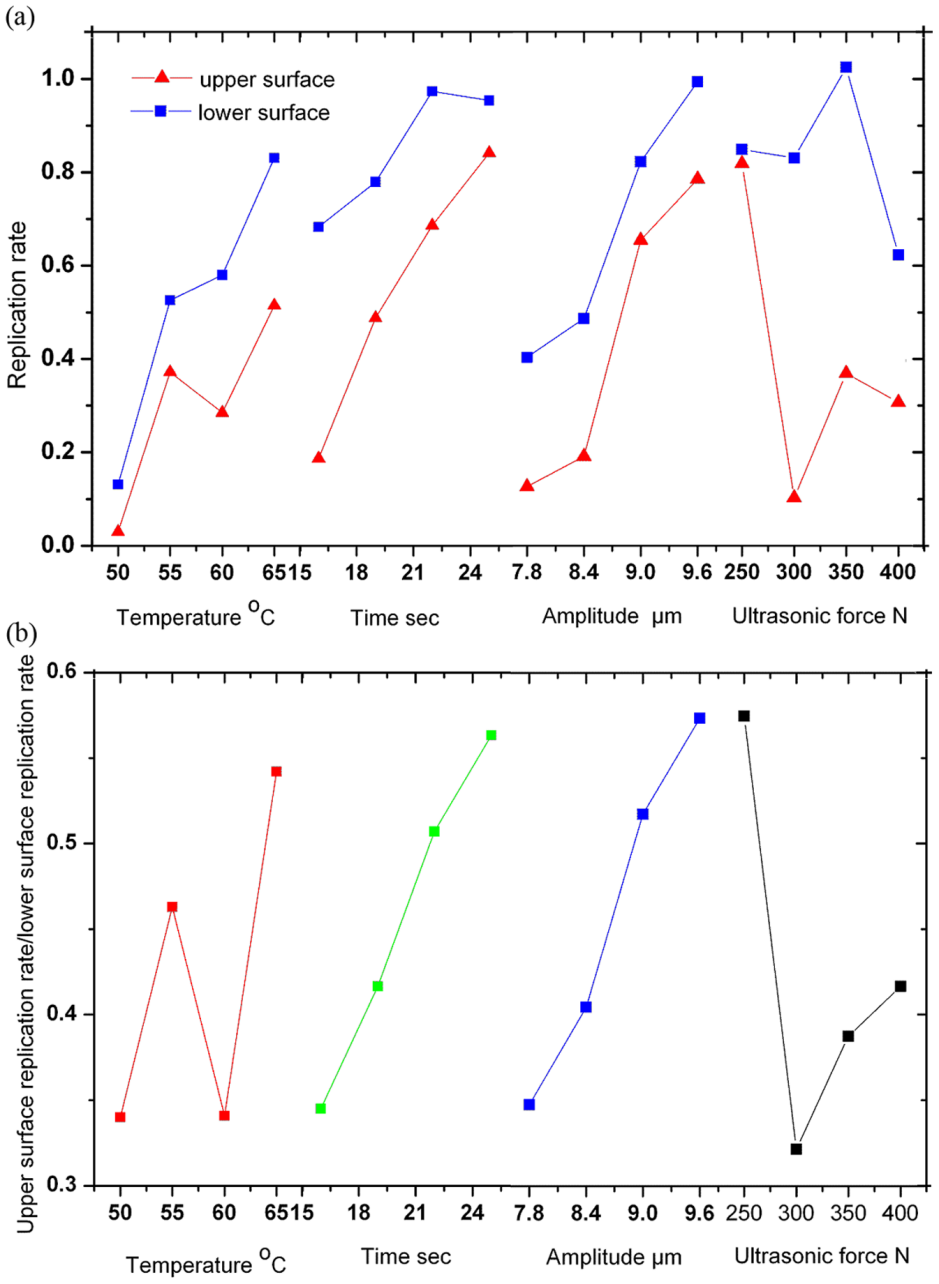
The relationship between embossing parameters (a) replication rate; (b) replication uniformity.

The analysis of variance (ANVA) of the replication depth and the uniformity on both sides of the PMMA substrates was conducted and the results are listed in Table 3. The different factor levels of the embossing parameters are shown in Fig. 6.

**Table 3:** The analysis of variance results of embossing parameters.

Parameters	F-test value		
	Replication depth on the upper surface	Replication depth on the lower surface	Uniformity of the replication on both sides of PMMA substrate
Thermal assisted temperature	1.878	5.270	0.421
Ultrasonic amplitude	4.060	4.030	1.840
Ultrasonic time	1.899	0.647	2.864
Ultrasonic force	1.031	0.422	2.339

The uniformity of the replication on both sides is expressed as:
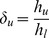
(1)Where *h_u_* is the depth of the microstructure on the upper surface, *h_l_* is depth of the microstructure on the lower surface.

**Figure 7:**
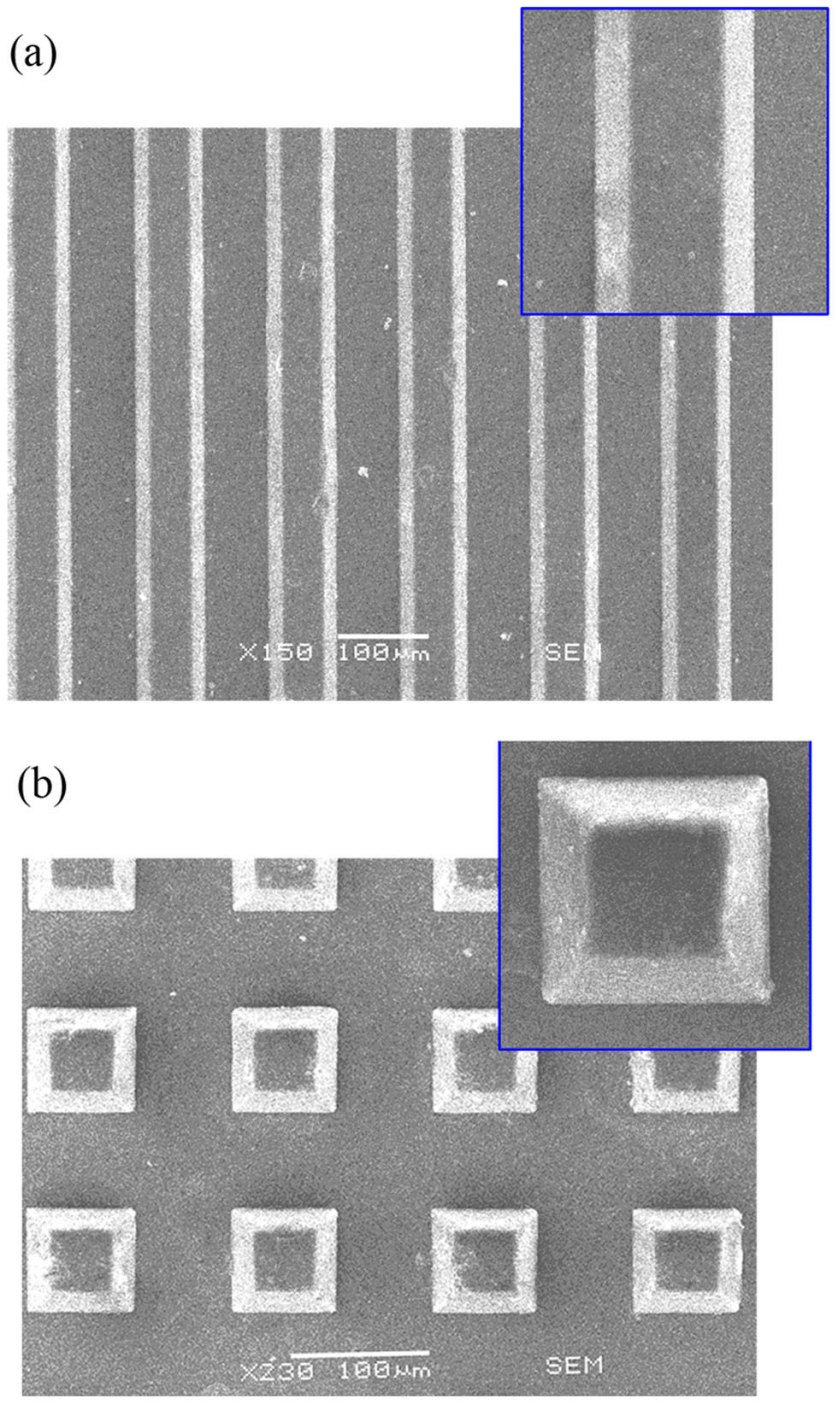
SEM images of embossed microstructure (a) longitudinal-stripe style; (b) checked style.

The replication results are shown in Fig. 7.

The ultrasonic amplitude has the largest influence on the replication depth on the upper surface followed by ultrasonic time. The larger the amplitude, the faster the heat generates. Ultrasonic time plays the second role in the embossing process, because the PMMA substrate is a poor heat conductor. A longer ultrasonic time is helpful to accumulate enough heat to soften the polymer and form the microstructures.

Unlike the upper surface replication, thermal assisted temperature has the most important effect on the lower surface followed by the ultrasonic amplitude. Since the lower surface directly contacted the hot plate and the silicon mold, the temperature gap to *Tg* reduced to about 40–60°C, while this gap was 60–80°. on the upper surface.

The uniformity of the replication on both sides of the PMMA substrates is majorly influenced by ultrasonic time, then ultrasonic force. As mentioned above, a longer ultrasonic time is helpful to accumulate heat, especially on the upper surface. For commonly, the temperature rising speed on the upper surface is lower than that on the lower surface. The larger ultrasonic force contributes to an even distribution of the stress at the interfaces of PMMA substrate and molds, which promotes the equivalent of the stress at contact area, softenes the polymer and creates an even replication.

### Influence of the thickness of the PMMA substrate

The thickness of the polymer substrate also affects the embossing results. In 1 mm thickness PMMA substrates replication, the ultrasonic embossing would be overheated when the ultrasonic amplitude reached 9.6 µm. The mold plunged into the polymer and made it difficult to demold. Using lower thermal assisted temperature, smaller amplitude, and the shorter ultrasonic time, the satisfactory replication results could also be reached, as shown in Table 4. Compared to No. 10 and 11 experiments in Table 2, the experiments were employed 2 mm PMMA substrates. The thermal assisted temperature and the ultrasonic amplitude had the same value, while the ultrasonic time and the ultrasonic force for 1 mm PMMA were equal or reduced. The replication rate for 1 mm PMMA was ranged from 97.1% to 99.9%, which, ranged from 2.7% to 55.6% for the 2 mm PMMA.

**Table 4:** Replication results of 1 mm PMMA substrate.

Thermal assisted temperature (°C)	Ultrasonic amplitude (µm)	Ultrasonic time (s)	Ultrasonic force (N)	Depth of the micro structures on PMMA substrate (µm)	
				Upper surface	Lower surface
60	8.4	17	350	20.73	20.72
60	9.0	14	350	21.28	20.68

**Figure 8:**
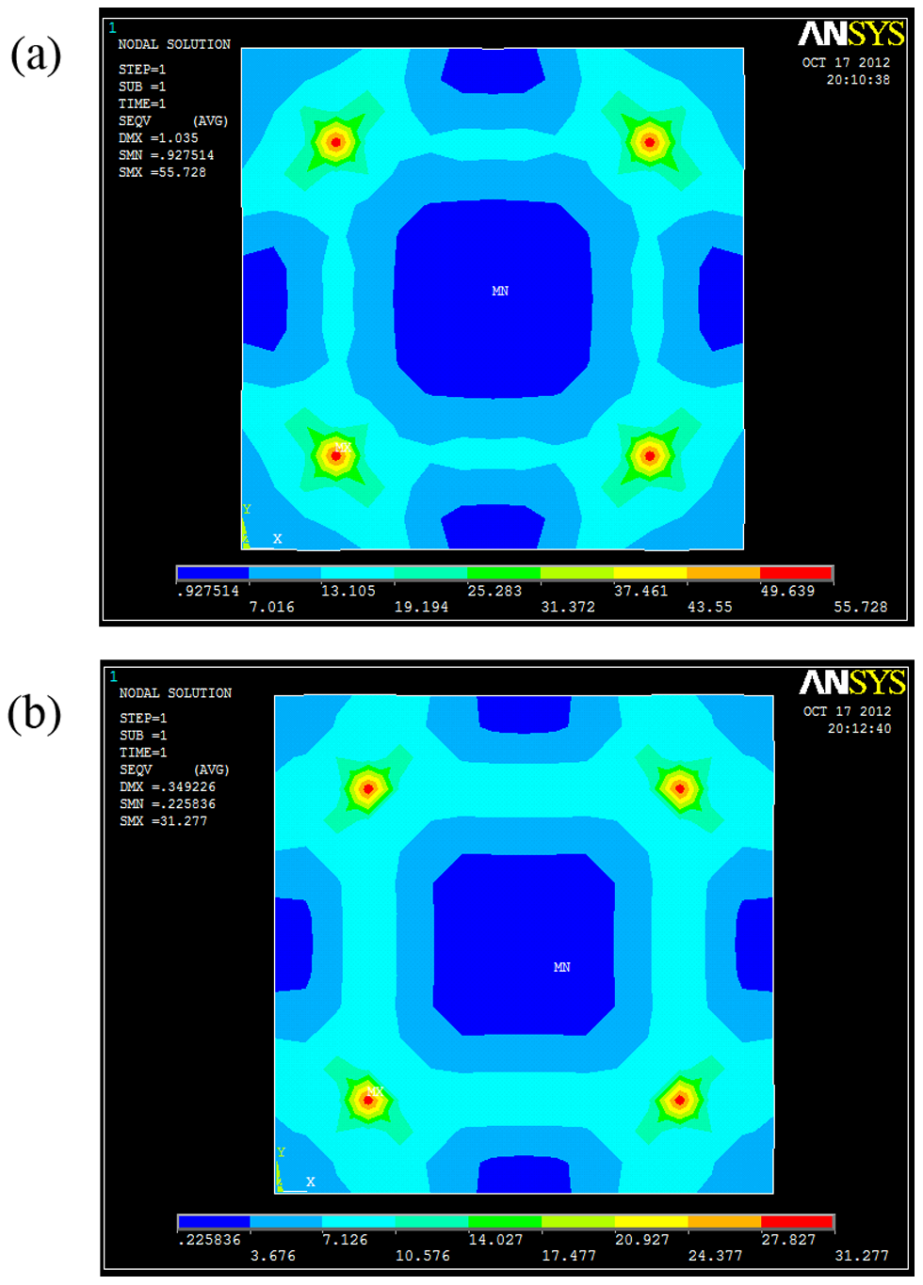
Stress distribution in the substrates with different thickness(a) 1 mm PMMA substrate; (b) 2 mm PMMA substrate.

The possible reason is that the stress for 1 mm was larger than 2 mm PMMA substrate under the same ultrasonic force. ANSYS was used to do the finite element analysis. The pattern was longitudinal-stripe style mold No. 2 in Table 1. The model was meshed by SOLID185 elements, the size of the analysis domain was 32×32×2 mm^3^, the patterned area was 10×10×2 mm^3^. The lower surface of the mold was fully fixed, and a force with the value of 400 N was applied on the upper surface of the PMMA substrate. Static analysis was conducted to study the stress under different substrate thickness. The highest stress for 1 and 2 mm PMMA substrates under the same ultrasonic force were 55.73 and 31.28 MPa, respectively, as shown in Fig. 8. Thus, the stress for 1 mm was higher than 2 mm PMMA substrate, which can benefit the heat generation under the same ultrasonic force. Though the ultrasonic force has the smallest influence among the four processing parameters in orthogonal array experiments, such a large stress increment can also accelerate the heat generation.

### Influence of the density and style of the microstructures

The density and style of the microstructures on the silicon molds also affect the replication results. Four kinds of experiments were conducted. The first was the silicon mold on the upper and lower surface had different density, show in Table 5, as Group A and B. The second, third and fourth kinds were based on the same mold density on the upper and lower surfaces by using different pattern style. The second was using longitudinal-stripe style mold, as Group C and D; the third was diagonal-strip style mold, as Group E and F; the fourth was using checked style mold, as Group G. The embossing parameters were selected based on the replication results of the orthogonal array experiments, thus the parameters in No. 9, 11, and 14 in Table 2 were selected. The detailed experiments parameters and results are listed in Table 5.

**Table 5:** The embossing parameters and replication depth with different molds.

Mold	Experiments number	Thermal assisted temperature (°C)	Ultrasonic amplitude (µm)	Ultrasonic time (s)	Ultrasonic force (N)	Depth of the micro structures on PMMA substrate (µm)	
						Upper surface	Lower surface
Group A (upper mold No. 2, lower mold No. 3)	A_1_	60	8.4	22	350	13.02	20.78
	A_2_	60	7.8	25	300	20.68	20.40
	A_3_	65	9.0	22	250	20.46	20.79
Group B (upper mold No. 2, lower mold No. 1)	B_1_	60	8.4	22	350	0.94	0.14
	B_2_	60	7.8	25	300	3.47	1.30
	B_3_	65	9.0	22	250	20.61	20.81
Group C (upper mold No. 1, lower mold No. 1)	C_1_	60	8.4	22	350	0.35	0.68
	C_2_	60	7.8	25	300	0.21	0.47
	C_3_	65	9.0	22	250	0.40	15.10
	C_4_	65	9.6	25	400	21.01	20.79
Group D (upper mold No. 3, lower mold No. 3)	D_1_	60	8.4	22	350	0.71	19.85
	D_2_	60	7.8	25	300	0.23	11.18
	D_3_	65	9.0	22	250	20.76	20.80
Group E (upper mold No. 4, lower mold No. 4)	E_1_	60	8.4	22	350	0.20	4.28
	E_2_	60	7.8	25	300	0.04	0.83
	E_3_	65	9.0	22	250	6.54	17.97
Group F (upper mold No. 5, lower mold No. 5)	F_1_	60	8.4	22	350	0.83	10.4
	F_2_	60	7.8	25	300	0.84	3.41
	F_3_	65	9.0	22	250	20.55	20.61
Group G (upper mold No. 6, lower mold No. 6)	G_1_	60	8.4	22	350	0.07	0.29
	G_2_	60	7.8	25	300	0.25	0.68
	G_3_	65	9.0	22	250	0.30	15.34
	G_4_	65	9.6	25	400	21.01	21.17

The width and pitch of each mold was listed in Table 1.

In Group A, the lower mold had lighter pattern density, so the lower surface is easier to reach high replication rate. It can still over 95% (Experiment No. A_1_) even at the amplitude of 7.8 µm. In Group B, the lower mold had heavier pattern density, thus the replication rate on the upper surface better than that on the lower surface (Experiment No. B_1_ and B_2_) until both sides accumulated enough heat to form the microstructures (Experiment No. B_3_). This phenomenon indicates that uniformity on both sides of the PMMA substrate could be improved by optimizing the pattern density and the embossing parameters, and the operation window for high quality replication could be widened, too.

In Group C and D, more energy was needed to reach a high replication rate with the increase of the microstructure density. For example, in Group C, from experiment No. C_1_ to C_3_, none of the depth reached 20 µm. The replication rate reached near 95% (Experiment No. C_4_) when the ultrasonic amplitude increased to 9.6 µm and the ultrasonic time reached 25 s. On the contrary, in Group D, it was easier to get high replication rate for the low density of the microstructures.

Group E and F were to replicate the diagonal-stripe style microstructures. The replication results were generally accorded with the results in Group C and D, especially in high replication rate experiments.

Checked style microstructures were replicated in Group G. The volume of the microstructures was the same as that in the orthogonal array experiments. The concave molds were used in Group G which is different from the convex molds used in the orthogonal experiments. Comparison to the orthogonal experiments, the replication rates in experiments G_1_ to G_3_ were lower, so the concave mold was difficult to replicate.

The experiments results indicate that the surface topography of the PMMA substrates also had significant influence on embossing in low replication rate. However, this influence decreased when the replication rate was over 95%. The possible reason is that the volume of the softened polymer increased with the replication rate approaching 100%. The fluidity of the polymer improved, thus the influence of the surface topography reduced.

When the replication rate approached 100%, an uncommon phenomenon appeared. Sometimes, the upper surface replication rates were a little higher than the lower surface, such as in A_1_ and C_4_. The possible reason is that the volume of the softened polymer increased on the upper surface when the replication is to complete. This portion of the polymer consumed ultrasonic energy, which reached the lower surface reduced. The upper surface had more energy than the lower surface in the final stage of the embossing. Sometimes, the replication rate on the upper surface can exceed the lower surface. This is accorded that a long ultrasonic time helps reach uniform replication on both sides of the polymer substrates.

## Conclusions

Double-side ultrasonic embossing was studied in this paper, and silicon molds were applied in ultrasonic embossing. In order to reduce the risk of damaging the molds and improve the replication uniformity on both sides of the polymer substrates, thermal assisted ultrasonic embossing method was proposed.

The influence of the thermal assisted temperature and ultrasonic parameters were studied using orthogonal array experiments. On the upper surface, the ultrasonic amplitude was the most significant parameters followed by ultrasonic force. On the lower surface, the thermal assisted temperature had the remarkable influence, then ultrasonic amplitude. And ultrasonic force was the most important parameter to get uniform replication on both sides of the substrate. The replication rate reached 97.5% on both sides of the PMMA substrate using the optimized parameters, with the cycle time less than 50 s.

The thickness of the PMMA substrate influenced the replication results too. According to the finite element analysis, a thinner substrate had higher stress distribution, and easier to reach high replication rate.

Furthermore, the impacts of the microstructure densities and styles were studied. The experimental results were shown that the concave mold was difficult to replicate, and heavy density microstructure needs more ultrasonic energy to replicate. Properly arrangement the different density molds on upper surface and lower surface can widen the operation window getting uniformity replication on both sides of the polymer substrates.
